# Perivascular fat attenuation index measured by coronary computed tomography angiography as a tool for assessment of ischaemia-causing lesions: a case report

**DOI:** 10.1186/s12872-023-03177-z

**Published:** 2023-03-18

**Authors:** Shuichi Okamoto, Junji Mochizuki, Hiroaki Matsumi, Katsushi Hashimoto, Akira Nikaido, Yoshiki Hata

**Affiliations:** Department of Cardiovascular Medicine, Minamino Cardiovascular Hospital, 1-25-1 Hyoue, Hachiouji, Tokyo 192-0918 Japan

**Keywords:** Spontaneous coronary artery dissection, Perivascular fat attenuation index, Coronary computed tomography angiography, Coronary artery disease

## Abstract

**Background:**

It can be difficult to diagnose coronary artery disease in patients with acute coronary syndrome if coronary angiography does not identify stenosis. Coronary inflammation, which can contribute to the pathogenesis of coronary artery disease and acute coronary syndrome, can be quantified using the perivascular fat attenuation index. Furthermore, the perivascular fat attenuation index is a marker for all-cause mortality, cardiac-related mortality and impaired global coronary flow reserve.

**Case presentation:**

Here we report a case of a patient presenting with symptoms of acute coronary syndrome. The patient had hypokinesis of the lateral-posterior wall of the left ventricle, decreased myocardial perfusion in the posterior wall myocardium and elevated myocardial troponin-T and creatine phosphokinase levels. However, coronary computed tomography angiography did not identify arterial stenosis. The patient did have an increased perivascular fat attenuation index, indicating coronary inflammation. Moreover, the fat attenuation index was higher around the left circumflex artery than around the right coronary artery or left anterior descending artery. Intravascular ultrasonography identified an intramural haematoma, leading to a diagnosis of type 3 spontaneous coronary artery dissection in the left circumflex artery.

**Conclusions:**

Perivascular fat attenuation index may be a useful tool to help identify and localise disease-causing lesions, and to direct further testing to confirm a diagnosis of spontaneous coronary artery dissection in acute coronary syndrome patients without significant arterial stenosis.

## Background

The diagnosis of coronary artery disease (CAD) is often difficult to make in patients with symptoms of acute coronary syndrome (ACS) if coronary angiography does not show severe stenosis of the coronary arteries. In such cases, measurement of the perivascular fat attenuation index (FAI) may help identify coronary inflammation and direct further diagnostic testing to identify CAD.

Epicardial adipose tissue, including the perivascular fat around coronary arteries, can secrete pro-inflammatory adipokines [1, 2]. Inflammation is known to contribute to the pathogenesis of CAD and ACS [1]. Furthermore, inflamed cardiac vessels can also release cytokines that alter the function and appearance of perivascular fat [3].

Perivascular FAI is a validated non-invasive marker for coronary inflammation [3, 4]. It is calculated by using standardised computed tomography angiography (CTA) images to assess changes in perivascular fat around the right coronary artery (RCA), left anterior descending artery (LAD) and left circumflex artery (LCX) [3, 4]. A perivascular FAI higher than − 70.1 Hounsfield units (HU) can predict all-cause mortality and cardiac-related mortality after adjustment for age, sex and other risk factors [4]. Moreover, perivascular FAI is associated with impaired global coronary flow reserve in patients with stable coronary artery disease [5] and has been reported in patients with spontaneous coronary artery dissection (SCAD) [6] and vasospastic angina [7]. Therefore, perivascular FAI may help identify at-risk patients who present with ACS but without significant coronary artery stenosis.

Here, we present a case of a patient with signs of ACS but with no significant coronary artery stenosis. We used CCTA images to identify increased perivascular FAI and intravascular ultrasonography to diagnose SCAD in the LCX of this patient.

## Case presentation

**Fig. 1:**
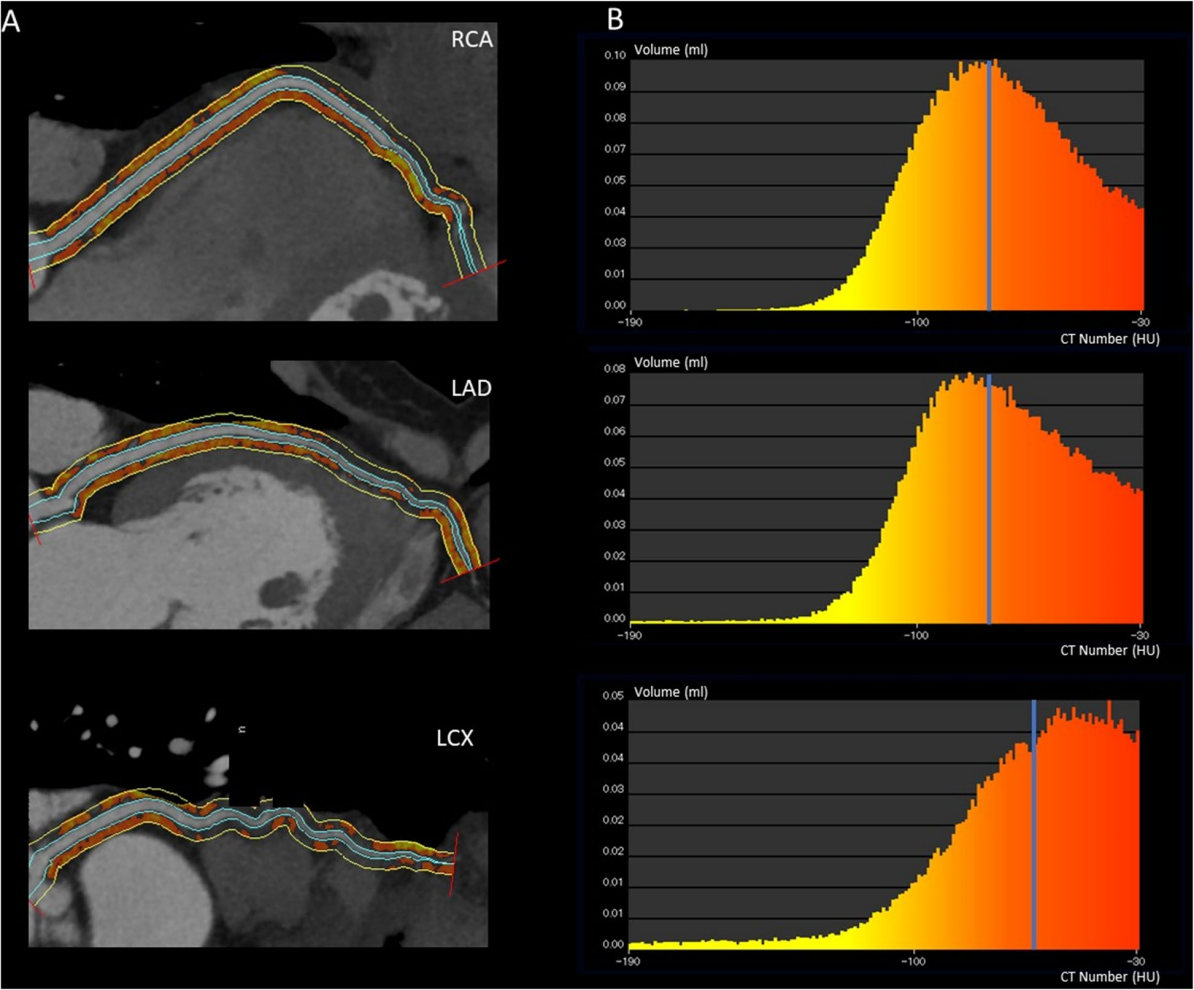
Perivascular FAI analysis of the RCA, LAD and LCX. **A** Colour map indicating CT results—red indicates a higher CT number, and yellow indicates a lower CT number. **B**. FAI analysis. Histograms of voxel CT attenuations within the volume of interest. The median CT attenuation range was: − 190 to − 30 HU. FAI, fat attenuation index; RCA, right coronary artery; LAD, left anterior descending artery; LCX, left circumflex artery; CT, computed tomography

A 49-year-old woman with the clinical characteristics of ACS was referred to our hospital. She had a 4-day history of chest pain. A 12-lead electrocardiogram (ECG) showed negative T waves in leads II, III and aVF. Transthoracic echocardiography (TTE) showed hypokinesis of the lateral-posterior wall of the left ventricle. The patient’s myocardial troponin-T and creatine phosphokinase (CPK) isoenzyme levels were elevated (1800 ng/mL and 229 U/L, respectively). Coronary computed tomography angiography (CCTA) images showed decreased myocardial perfusion in the posterior wall myocardium; however, they did not show significant coronary artery stenosis. Therefore, we measured the perivascular FAI for all three coronary arteries to quantify coronary inflammation. Using a previously described and validated method^4^, we traced a 3-mm width of the coronary adventitia along the total length of the three main coronary arteries in the CCTA images. Our measurements differed from those of the original report because we traced the proximal 40-mm segments of all three major epicardial coronary vessels. The total length of the coronary artery was approximately 155 mm for all three branches. We defined perivascular fat as adipose tissue surrounding the vessel wall within a distance equal to the diameter of the vessel [4]. As previously reported [3], we identified perivascular FAI using the attenuation histogram of perivascular fat, within the range − 30 to − 190 HU, as measured by CCTA (Fig. 1A).

**Fig. 2:**
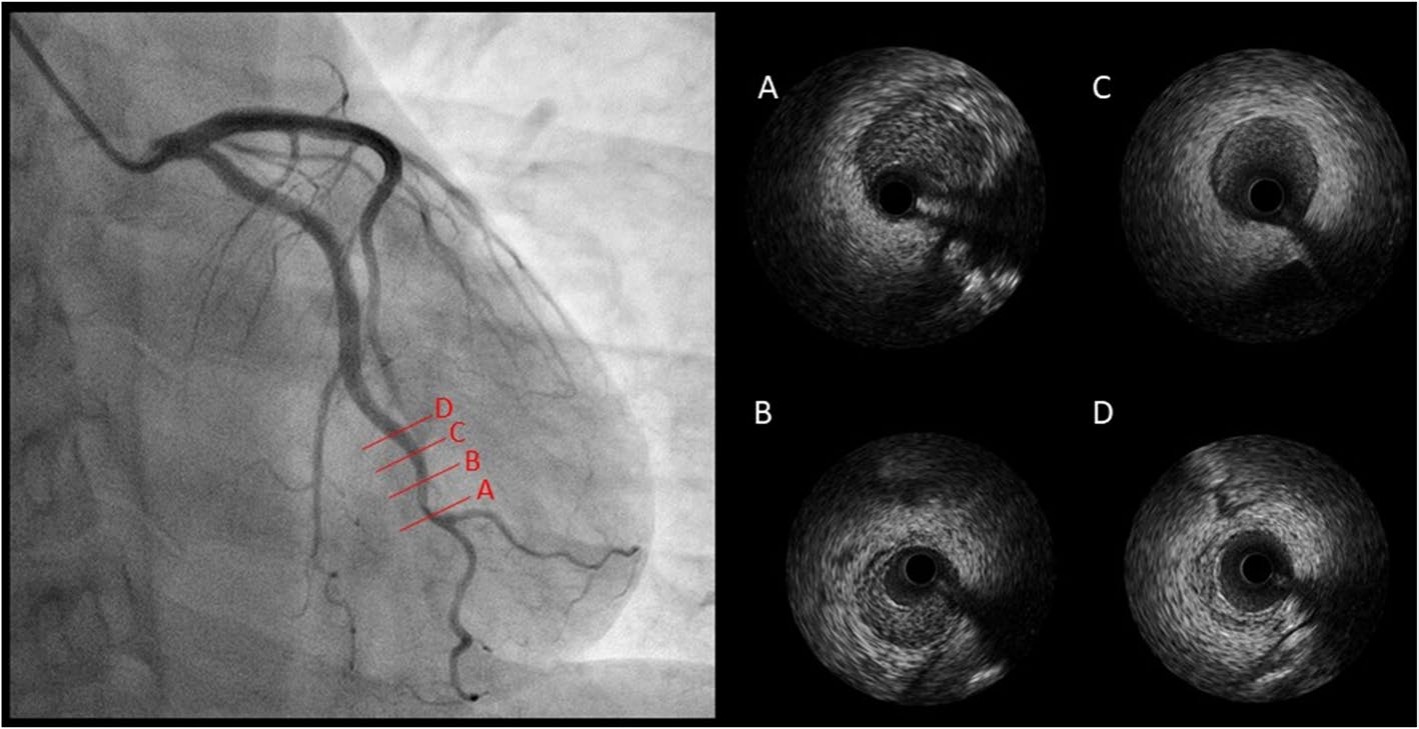
SCAD lesion imaged at four levels in the LCX. The red lines **A**, **B**, **C**, **D** in the left panel correspond with the images in the right panel. We diagnosed an intramural haematoma with observed heterogeneity on IVUS. SCAD, spontaneous coronary artery dissection; LCX, left circumflex artery; IVUS, intravascular ultrasonography

We found significantly higher FAI around the LCX (median value of − 57 HU) than around the LAD and RCA (median FAIs − 73 HU and − 74 HU, respectively, Fig. 1B). Coronary angiography identified moderate stenosis in the distal segment of the LCX (Fig. 2). Intravascular ultrasonography was used to evaluate lesion morphology and identified an intramural haematoma in the LCX, consistent with type 3 SCAD (Fig. 2). Table 1 shows a timeline of the patient’s course.

**Table 1:** Patient Timeline

Main complaint: chest pain of 4 days’ duration
ECG: negative T wave in leads II, III and aVF
TTE: hypokinesis at the lateral-posterior wall of the left ventricle
Laboratory data: troponin-T: 1.800 ng/mL; CPK: 229 U/L
CCT: no significant coronary artery stenosis but significantly higher FAI score in the LCX vs the LAD and RCA
Cardiac catheterisation: moderate stenosis in the distal segment of the LCX
IVUS: coronary dissection in the LCX
Clinical course: Uneventful with a conservative medical management strategy

*ECG* Electrocardiogram, *TTE* Transthoracic echocardiography, *CPK* Creatine phosphokinase, *CCT* Cardiac computed tomography, *FAI* Fat attenuation index, *LCX* Left circumflex artery, *LAD* Left anterior descending artery, *RCA* Right coronary artery, *IVUS* Intravascular ultrasonography

## Discussion and Conclusions

Coronary angiography does not identify coronary artery stenosis in a subset of patients presenting with ACS symptoms. In the case presented in this report, we performed CCTA to identify the cause of the patient’s chest pain, and although we found decreased myocardial perfusion in the posterior wall myocardium, we did not find significant stenosis in the coronary arteries. However, the patient’s decreased myocardial perfusion and elevated myocardial troponin-T and CPK levels indicated that myocardial ischaemia was the likely cause of the chest pain. Therefore, we evaluated perivascular FAI to quantify coronary inflammation. The patient’s elevated perivascular FAI around the LCX suggested that an adverse event had occurred in the coronary arteries, most likely in the LCX, so we performed intravascular ultrasonography, leading to a definitive diagnosis of SCAD.

SCAD is defined as separation of arterial wall layers, creating a false lumen without prior trauma or atherosclerosis [6]. SCAD is implicated in up to 35% of cases of myocardial infarction in women under 50 years of age [8]. In cases of SCAD, intramural haematoma rather than atherosclerotic plaque can obstruct coronary blood flow [9]. We found an intramural haematoma in the LCX of our patient. These results suggest that SCAD was the cause of myocardial ischaemia and chest pain in this patient.

Increased perivascular FAI is a risk factor for all-cause and cardiac-related mortality. The Cardiovascular RISk Prediction using Computed Tomography (CRISP-CT) study mapped perivascular fat attenuation in the proximal RCA, the LAD and the LCX in two cohorts of patients from Germany and the US [4]. High perivascular FAI around all three arteries predicted all-cause mortality [4]. However, only high perivascular FAI around the proximal RCA and LAD predicted cardiac-related mortality [4]. The study identified a cutoff of − 70 HU or higher as optimal for predicting cardiac and all-cause mortality [4]. Perivascular FAI is also higher in patients with vasospastic angina than in those with non-vasospastic angina [7].

In contrast, the link between inflammation, perivascular FAI and SCAD remains unclear. Perivascular fat attenuation has been reported in two SCAD patients [6]. Conversely, a larger study of 11 SCAD patients and 27 controls found no difference in epicardial fat or in perivascular fat attenuation between the two patient groups [10].

Here, we present a case of an ACS patient who did not show significant coronary arterial stenosis, but who did have a higher FAI in the LCX than that in the RCA or LAD. The high FAI helped us to direct further imaging to the LCX and to diagnose SCAD in this vessel. The data presented in this report is from a single case and, therefore, further studies are required to confirm these findings.

This case shows that using CCTA to measure perivascular FAI in the coronary arteries may be a useful tool to direct further testing and confirm a diagnosis of SCAD in ACS patients, even if no obvious atherosclerotic lesion is found in the coronary arteries.

## Data Availability

All data generated or analysed during this study are included in this published article.

## Abbreviations

ACS: Acute coronary syndrome
CAD: Coronary artery disease
CCTA: Coronary computed tomography angiography
CPK: Creatine phosphokinase
CRISP-CT: Cardiovascular RISk prediction using computed tomography
CTA: Computed tomography angiography
ECG: Electrocardiogram
FAI: Fat attenuation index
HU: Hounsfield units
LAD: Left anterior descending artery
LCX: Left circumflex artery
RCA: Right coronary artery
SCAD: Spontaneous coronary artery dissection
TTE: Transthoracic echocardiogram
